# Penicillin allergies: referral and management practices of anesthesiologists

**DOI:** 10.1186/1710-1492-10-S2-A20

**Published:** 2014-12-18

**Authors:** V Jain, N Joshi, M Sidhu, C Kalicinsky, T Pun

**Affiliations:** 1 Allergy & Clinical Immunology, University of Manitoba, Winnipeg, MB, R3T 2N2, Canada; 2 Department of Internal Medicine, Memorial University of Newfoundland, St. John’s, NL, A1B 3X9, Canada; 3 Department of Family Medicine, University of Western Ontario, London, ON, N6A 3K7, Canada

## Rationale

Penicillin and other beta-lactams are the most commonly used antibiotics due to their narrow spectrum of activity, low cost and safety profile. However, an “allergy” to Penicillin is also the most commonly reported allergy. Approximately, 5-10% of all patients self-report an allergy to Penicillin and of these <10% are found to have true IgE mediated allergy on skin testing.

Numerous studies have confirmed the usefulness and strong negative predictive value (>99%) of skin testing to rule out true IgE mediated Penicillin allergy. Less than 10% of patients with a history of penicillin allergy are found to be actually allergic to penicillin on skin testing. Despite this, most physicians forgo further investigations in favor of the usage of alternative antibiotics.

## Methods

A questionnaire was designed to evaluate the referral practices of Anesthesiologists for a presumed Penicillin allergy.

The preliminary study was administered as a semi-structured interview to Anesthesiology Staff Physicians and Senior Residents at Memorial University of Newfoundland. The responses were analyzed using recursive abstraction

## Results

89.5% of respondents have never referred patients for evaluation of drug allergy, although, an equal number felt a referral would be helpful. However, 47.3% said they have verbally communicated to their patients that they should speak to their Family Doctor for work up of their allergy. 21.1% of participants felt time constraint was a barrier to creating a referral; another 15.8% felt that this was the responsibility of another physician (Surgeon or Family Doctor). An additional 26.3% did not comment on barriers but stated they would just give an alternative medication rather than refer. Another 15.8% mentioned that surgery is generally imminent and would not delay surgery to a referral. All participants stated they would choose an alternative antibiotic in the case of a history of penicillin allergy.

## Conclusion

Carrying a presumed diagnosis of penicillin ‘‘allergy’’ has significant consequences on the health care system and patient outcome. Anesthesiologists in our study do inquire about specifics of allergy history, however, the referrals are virtually non-existent. As a result, anesthesiologists are prescribing more expensive antibiotics, which have higher potential for emergence of antibiotic resistance. Our future plans are to complete data collection at other centers and to develop an intervention to improve referral practices and study its impact.
